# Prolonged Disease Stability with Durvalumab and Tremelimumab Rechallenge Following Prior Immune Checkpoint Inhibitors: A Case Report

**DOI:** 10.32604/or.2026.083193

**Published:** 2026-08-13

**Authors:** Waseem Abdelrahim, Ebtesam Al-Najjar, Seif El Beheary, Abdullah Esmail

**Affiliations:** 1 Michael E. DeBakey HS for Health Professions, Houston, TX, USA; 2 Section of GI Oncology, Houston Methodist Neal Cancer Center, Houston Methodist Hospital, Houston, TX, USA; 3 College of Natural Sciences, University of Houston, Houston, TX, USA

**Keywords:** Advanced hepatocellular carcinoma (HCC), adrenal glands, rechallenge, Durva/Treme, case report

## Abstract

**Background:** Hepatocellular carcinoma (HCC) is the most common primary liver malignancy and remains a leading cause of cancer-related mortality worldwide due to frequent recurrence and early metastasis. While immune checkpoint inhibitor (ICPI)-based regimens have revolutionized the treatment landscape for advanced HCC, clinical evidence regarding the safety and efficacy of ICPI rechallenge following disease progression or severe immune-related adverse events (irAEs) remains sparse. This report describes a case of prolonged disease stability achieved through sequential immunotherapy using durvalumab and tremelimumab (Durva/Treme) after prior ICPI failure and high-grade toxicity. Case Description: A 65-year-old male with recurrent stage IV HCC and metastases to the adrenal glands and vertebral column was referred to our center after failing multiple lines of therapy, including atezolizumab/bevacizumab, sorafenib, and cabozantinib. Subsequent treatment with nivolumab plus ipilimumab was complicated by grade 3/4 immune-related hepatitis, necessitating a treatment hold and steroid intervention. Despite an initial response in hepatic lesions, imaging confirmed progression in the adrenal metastases. The patient was then transitioned to a rechallenge protocol with the STRIDE regimen (Durva/Treme). The patient completed 39 cycles of therapy over more than three years. Serial imaging demonstrated sustained stable disease (SD), and a significant reduction in alpha-fetoprotein (AFP) levels initially declined substantially during nivolumab/ipilimumab (Nivo/Ipi) therapy, rebounded prior to Durva/Treme initiation, and subsequently stabilized near baseline levels during ongoing Durva/Treme treatment. Notably, the rechallenge was well-tolerated with no recurrence of high-grade hepatotoxicity. **Conclusions:** This case demonstrates that ICPI rechallenge with Durva/Treme may provide durable disease control in selected patients with advanced HCC, even those with a history of severe immune-related toxicity. While the localized response was likely augmented by interval radiotherapy, the overall clinical course suggests that the STRIDE regimen may offer a feasible sequential immunotherapy option. These findings warrant further prospective investigation into the biological mechanisms of immune re-priming and the safety of immunotherapy sequencing in advanced oncology.

## 1 Introduction

The landscape of oncology has undergone a profound transformation over the last century, evolving from a reliance on localized interventions to a sophisticated era of precision medicine. Historically, cancer management was defined by the ‘three pillars’ of surgery, radiotherapy, and cytotoxic chemotherapy, which, while effective for certain stages, often struggled with systemic resistance and significant treatment-related morbidity. The paradigm shifted with the advent of targeted therapies and, most notably, the emergence of immunotherapy, which harnesses the host’s immune system to recognize and eradicate malignant cells. This evolution reflects a broader transition from non-specific cell-killing strategies to highly specific, biology-driven interventions that have significantly extended survival across multiple tumor types [1,2,3,4,5]. Within this historical trajectory, the development of immune checkpoint inhibitors (ICPIs) represents a pivotal milestone, particularly for recalcitrant malignancies like hepatocellular carcinoma (HCC), where traditional systemic options were historically limited.

Liver cancer is the sixth most prevalent cancer and the third leading cause of cancer-related mortality in the world. HCC, the most common primary liver malignancy, is an aggressive, chronic inflammation-associated cancer with poor long-term survival and high mortality rates [6]. Based on the Global Burden of Disease (GBD) study in 2021, there were 529,000 new cases of liver cancer and 483,000 related deaths globally [7]. The two major types of liver cancer are HCC and intrahepatic cholangiocarcinoma, along with less common types such as angiosarcoma, hemangiosarcoma, and hepatoblastoma [8]. HCC is the most common type, ranking as the seventh leading cause of cancer-related deaths in the United States (US) [9]. Nevertheless, HCC remains an aggressive malignancy, with high rates of post-resection recurrence and a 5-year survival rate below 20% [9].

The most frequent causes of HCC include chronic infections with hepatitis B or C viruses, nonalcoholic fatty liver disease (NAFLD), and alcoholic liver disease [10]. Treatment strategies for HCC are typically guided by the Barcelona Clinic Liver Cancer (BCLC) staging system. Surgical resection or orthotopic liver transplant (OLT) is the standard approach for early-stage HCC (BCLC stage A) and can be curative based on liver function status and tumor size [7,11]. BCLC stage B represents the intermediate stage and includes patients with multinodular disease, who are typically managed with locoregional therapies (LRTs). Patients with advanced, unresectable HCC characterized by portal vein invasion or distant metastases, preserved liver function (Child-Pugh Class A), and Eastern Cooperative Oncology Group (ECOG) performance status of ≤2 are classified as BCLC stage C. More than 80% of patients are diagnosed with an advanced-stage disease, making resection, ablation, or LRTs more challenging. These patients are primarily treated with systemic therapies. While most patients exhibit significant resistance to standard chemotherapy and radiotherapy; however, outcomes have improved significantly with the immunotherapy (IO) regimens [12,13].

Over the past decade, sorafenib, a multikinase inhibitor primarily targeting anti-vascular endothelial growth factor receptor 2 (VEGFR-2), and RAF kinase, was considered the standard first-line systemic treatment of advanced or metastatic HCC. Based on findings from the RFELECT trial, using a non-inferiority endpoint design, lenvatinib was recently approved as an alternative first-line treatment to sorafenib [14].

Recent advances in systemic therapy, particularly the use of immune checkpoint inhibitors (ICPI) in combination therapy, have played a critical role in systemic treatment, revolutionizing the management of advanced HCC [15]. The IMbrave150 trial demonstrated that the combination of atezolizumab and bevacizumab (Atezo/Bev) targeting programmed death ligand 1 (PD-L1) and anti-vascular endothelial growth factor (VEGF), significantly improved overall survival (OS) at 12 months compared to sorafenib. As a result, this combination has been approved as a first-line treatment and is recommended in the National Comprehensive Cancer Network (NCCN) treatment guidelines.

Another combination, durvalumab and tremelimumab (Durva/Treme), which inhibits anti-cytotoxic T-lymphocyte-associated antigen 4 (CTLA-4) and anti-PD-L1 pathways, also showed superior efficacy over sorafenib in the HIMALAYA trial, with a median OS of 16.4 months [15]. Both regimens are now recommended as first-line therapies for advanced HCC per BCLC guidelines [15].

Following first-line IO treatment, decisions on subsequent therapy are guided by clinical status, adverse effect profiles, and the available treatment options [16]. The effectiveness of further ICPI therapy in patients previously treated with ICPI remains uncertain due to a lack of prospective trial data [11]. However, positive outcomes from ICPI rechallenge have been observed in other solid tumors, supporting the need to explore this approach in HCC patients as well [17].

For patients who experience disease progression after treatment with Atezo/Bev, current guidelines suggest using tyrosine kinase inhibitors (TKIs) or ramucirumab as monotherapy. Some case series have reported that a combination of nivolumab and ipilimumab (Nivo/Ipi) may offer a second-line treatment option [18]. Despite therapeutic advances, outcomes in advanced unresectable HCC remain heterogeneous, underscoring the need for noninvasive biomarkers to predict disease progression. Pretreatment multiparametric MRI features, including tumor number and arterial-phase enhancement, have been shown to predict progression in patients treated with Lenvatinib plus transarterial chemoembolization, with radiomics further improving prognostic performance [13].

Here, we present a case of a patient with recurrent advanced-stage IV HCC with metastases to the adrenal glands and vertebral bones, who showed disease progression after receiving multiple lines of systemic therapy. The patient was subsequently rechallenged with an ICPI regimen, Durva/Treme, and has maintained stable disease for over two years to date. This case contributes to the emerging evidence supporting the potential role of sequential ICPIs strategies in advanced metastatic HCC. It demonstrates that rechallenging with a different ICPI combination may offer sustained clinical benefit, even in patients who have progressed on prior ICPI-based therapies. As a single-patient case report, this study was exempt from formal review by the Institutional Review Board (IRB) of Houston Methodist Hospital. The handwritten informed consent was obtained from the patient. Besides, this study was prepared according to the CARE case report guideline, and a CARE checklist was provided [19].

## 2 Case Presentation

**Figure 1:**
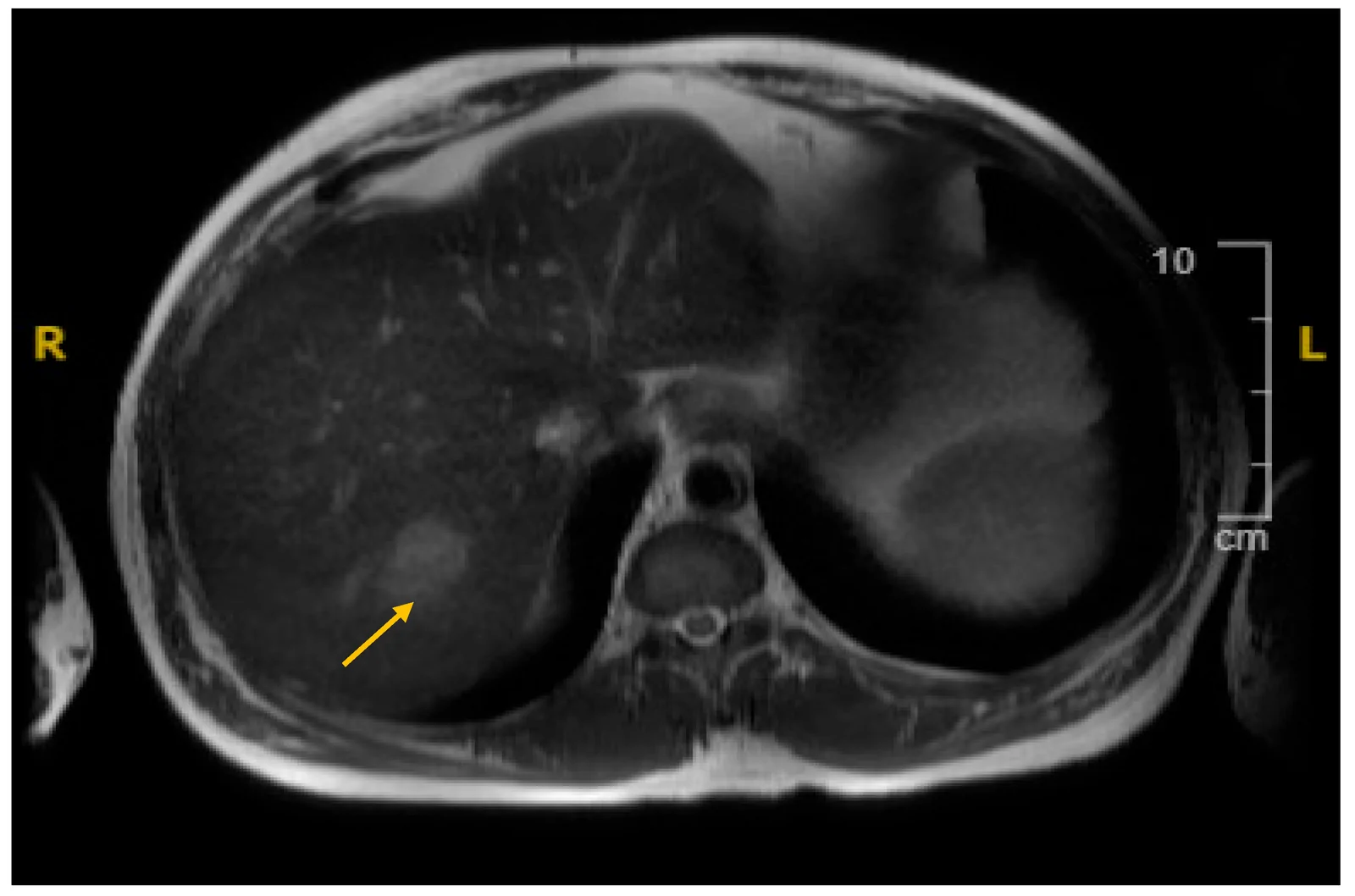
At diagnosis, there is a 4.0 cm lesion within segment 7 of the liver.

**Figure 2:**
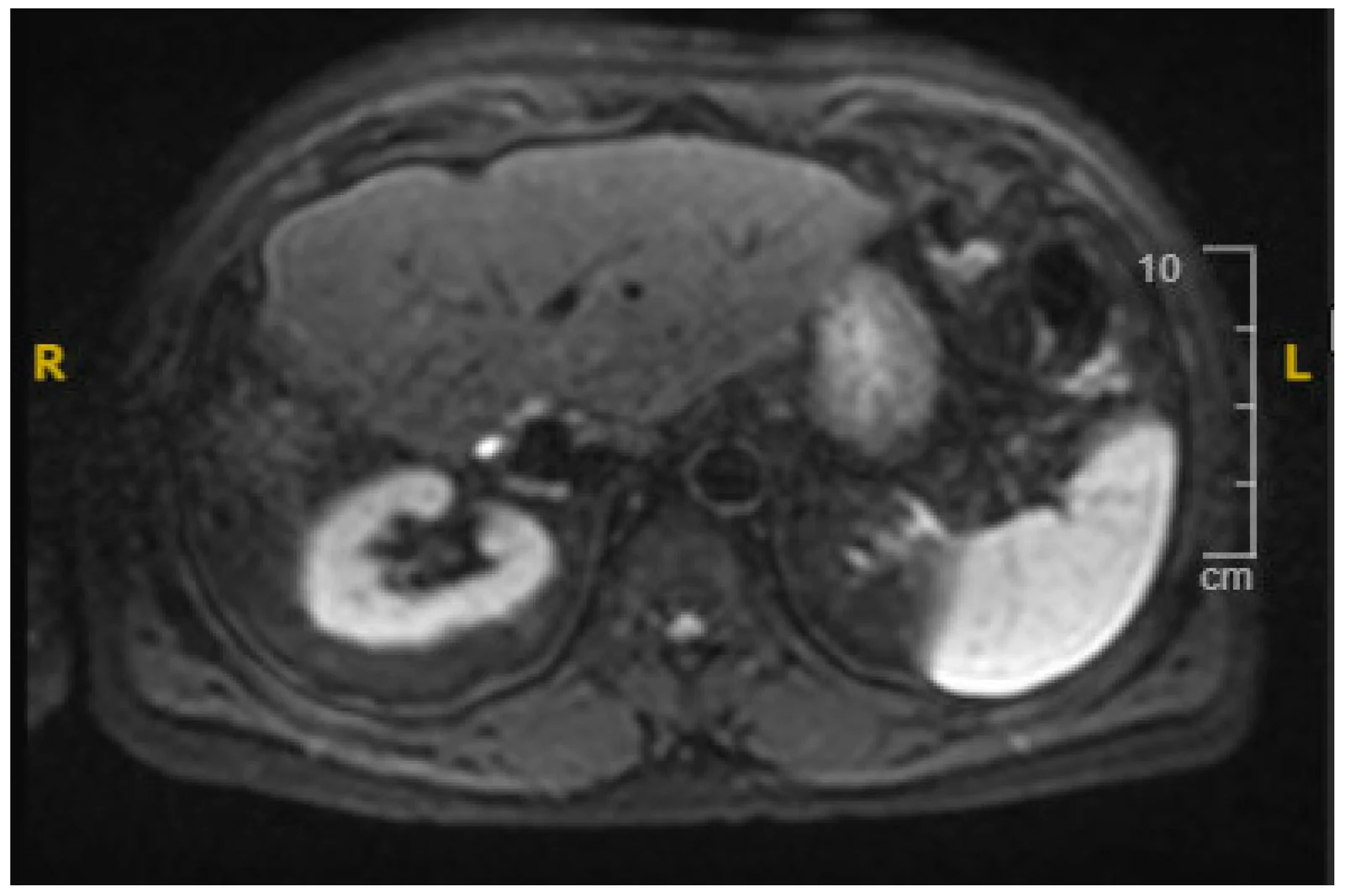
Post-hepatectomy follow-up MRI demonstrating stable postoperative hepatic changes without evidence of active intrahepatic recurrence at that time.

A 65-year-old male was diagnosed with HCC at an outside facility and was referred to Houston Methodist Neal Cancer Center (HMNCC) for further management. He was initially diagnosed in 2019 with stage II HCC, a 4.0 cm lesion within segment 7 of the liver (Fig. 1). Later he underwent a right hepatectomy (Fig. 2). Pathology revealed a moderately differentiated tumor grade 2 (G2) with negative surgical margins (pT2N0M0). Gene variant analysis revealed no mutations.

The patient had a history of chronic hepatitis C, which had been successfully treated with ledipasvir/sofosbuvir. His medical history was also significant for chronic obstructive pulmonary disease (COPD) not requiring oxygen supplements, insulin-dependent diabetes mellitus, hypertension (HTN), and esophageal varices without bleeding. His family history was notable for a sister diagnosed with esophageal cancer and a brother with colon cancer. Socially, he is a former smoker with a 25-year history of cigarette use, having quit in 2013. He denies current alcohol consumption but reported prior to social use, averaging 3–4 standard drinks per week.

One year after the initial diagnosis, a computed tomography (CT) scan revealed multiple enhancing hepatic masses. The dominant lesion in the left hepatic lobe measured 6.4 × 8.4 × 7.5 cm. and an additional possible 9 mm lesion in the remnant right hepatic region. These findings are consistent with disease recurrence. Additionally, a new mass was identified in the left adrenal gland, measuring 3.5 × 2.7 cm, which was also identified, confirming metastatic disease. Laboratory tests showed an elevated alpha-fetoprotein (AFP) level. Based on NCCN guidelines for Child-Pugh Class A patients, the patient was initiated on first-line palliative therapy with Atezo/Bev. Re-staging after cycle 8, using CT scan imaging and AFP levels, indicated the disease response to treatment. However, subsequent magnetic resonance imaging (MRI) revealed osseous metastases to the spine. The gastrointestinal oncology team recommended continuation of Atezo/Bev with the addition of radiation therapy (XRT) to the affected spinal region. The patient went on to complete 10 cycles. Despite this, follow-up imaging and rising AFP levels indicated continued disease progression. Based on this, the team decided to change the treatment regimen.

The patient was started on sorafenib as a second-line treatment. However, it was poorly tolerated, with the development of G3 mucositis and oral candidiasis, leading to treatment hold. Sorafenib was restarted at a reduced dose, but the patient’s symptoms persisted. Subsequently, cabozantinib was initiated at 40 mg orally daily as third-line treatment, but due to fatigue, nausea, and hand-foot syndrome (HFS), the dose was reduced to 20 mg daily, and supportive measures, including topical emollients and steroid cream to manage symptoms. The patient eventually self-discontinued treatment due to intolerance. A follow-up positron emission tomography (PET) scan revealed disease progression with bilateral adrenal metastases and multiple osseous lesions, leading to referral to HMNCC.

He was then started on nivolumab 1 mg/kg plus ipilimumab 3 mg/kg intravenously every 3 weeks as a fourth–line therapy. After two cycles, the AFP level decreased significantly. However, this course was complicated by G 3/4 immune-related hepatitis. Laboratory findings showed elevated liver enzymes: alanine aminotransferase (ALT) at 357 U/L, aspartate aminotransferase (AST) at 305 U/L, and bilirubin at 1.2 mg/dL. Nivo/Ipi was held, and high-dose steroid 1.5 mg/kg/day was initiated.

Follow-up MRI revealed progression in the bilateral adrenal glands, while the liver lesions demonstrated a positive response to the treatment. Given the prior good response to IO and the safer profile of Durva/Treme observed in the HIMALAYA trial particularly the lower rate of AST elevation compared to the Nivo/Ipi regimen in the CheckMate 040 trial, the decision was made to begin Durva/Treme. The patient was subsequently initiated on the STRIDE regimen consisting of tremelimumab 300 mg as a single priming dose plus durvalumab 1500 mg intravenously, followed by durvalumab 1500 mg every 4 weeks.

Re-staging MRI after five cycles of Durva/Treme showed stable disease (SD) in the liver and vertebral bone lesions, accompanied by a continuous decrease in AFP levels. The patient tolerated the regimen well, with the only reported side effect being oral thrush, successfully treated with nystatin mouthwash and a 7-day course of fluconazole. After ten cycles, imaging showed continued SD in the liver but slight progression in the right adrenal gland, for which the patient underwent stereotactic body radiation therapy (SBRT).

**Figure 3:**
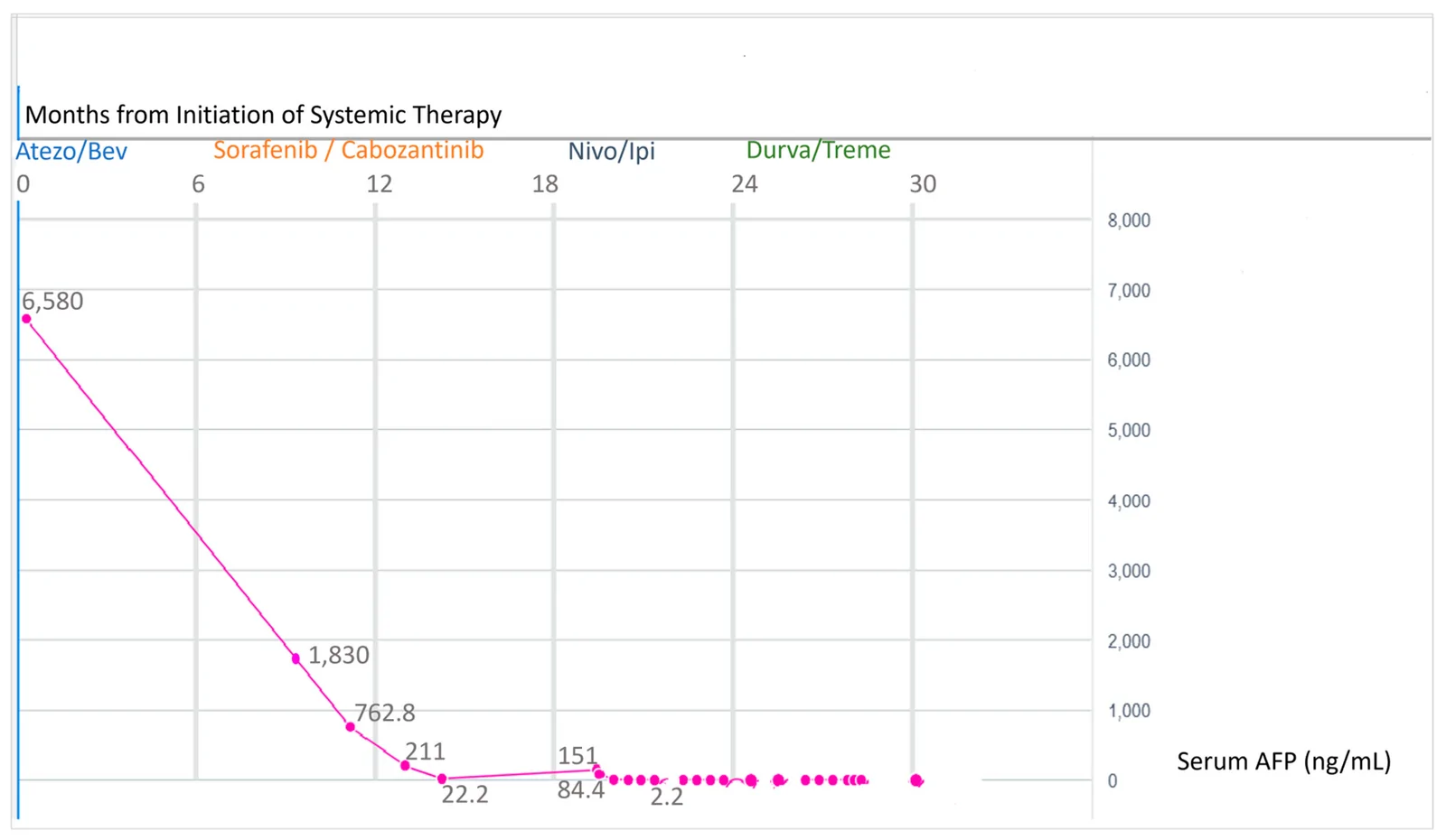
Serial alpha-fetoprotein (AFP) levels measured throughout the patient’s treatment course for advanced hepatocellular carcinoma (HCC). AFP levels were markedly elevated at baseline and demonstrated a substantial decline following immune checkpoint inhibitor therapy. Following transient fluctuations during disease progression and treatment transitions, AFP levels normalized and remained stable during ongoing durvalumab/tremelimumab (Durva/Treme) therapy, correlating with sustained radiographic stable disease. The *Y*-axis represents serum AFP levels (ng/mL), and the *X*-axis represents months from initiation of systemic therapy.

**Figure 4:**
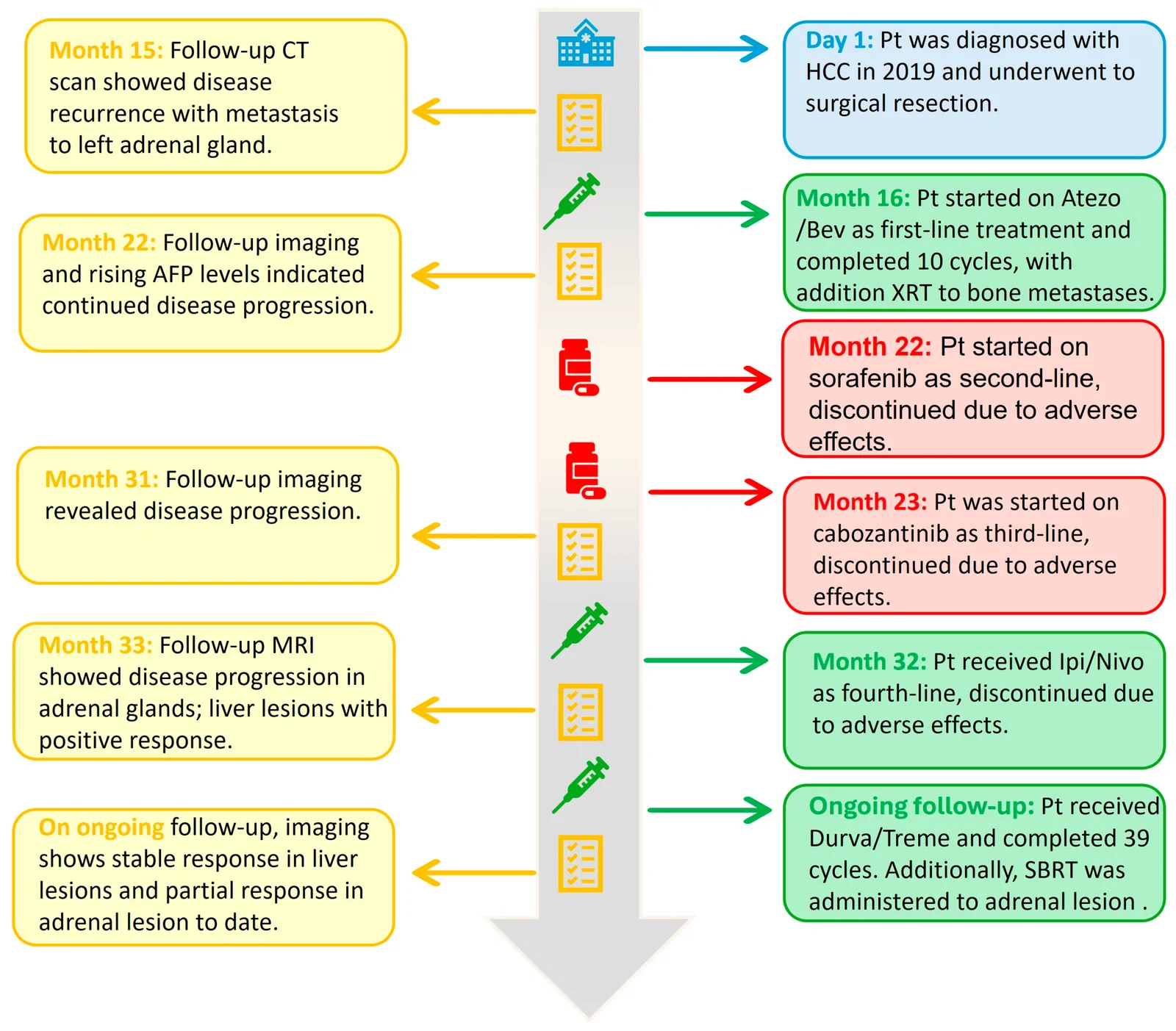
Timeline summarizing the patient’s clinical course from initial diagnosis of Hepatocellular carcinoma through multiple lines of systemic therapy. Following disease progression and treatment-related adverse effects with earlier therapies, the patient was rechallenged with durvalumab/tremelimumab (Durva/Treme), achieving sustained stable disease (SD) in the liver lesions and a partial response (PR) in the adrenal lesion. The patient remains on therapy with ongoing clinical benefit. CT, computed tomography; HCC, hepatocellular carcinoma; MRI, magnetic resonance imaging; XRT, external beam radiation therapy; SBRT, stereotactic body radiation therapy; AFP, alpha-fetoprotein; SD, stable disease; PR, partial response.

A follow-up scan after 18 cycles revealed a partial response (PR) in the right adrenal lesion, continued SD in liver lesions and bone metastases, and return of AFP levels to normal. Serial AFP levels during treatment are shown in Fig. 3. The patient has now successfully completed 39 cycles of Durva/Treme every 4 weeks over more than three years and continues to demonstrate SD with no immune-related adverse effects (irAEs). The timeline in Fig. 4 provides a detailed overview of a patient’s diagnosis and treatment course.

## 3 Discussion

This case report describes a patient initially diagnosed with HCC who underwent curative hepatectomy. The patient later developed recurrent liver lesions and metastases to the adrenal glands and vertebral bones, with disease progression following treatment with Atezo/Bev. The patient was intolerant to multiple TKIs, including sorafenib, cabozantinib, as well as Nivo/Ipi, due to severe irAEs. Rechallenge with a different ICPI combination, Durva/Treme, led to sustained disease control for over two years without further irAEs.

For patients with HCC who progress after first-line systemic treatment, evidence supporting ICPI rechallenge is limited [20]. Immune rechallenge is a therapeutic strategy that involves re-administering the same class of IO to patients with unresectable or metastatic cancer who previously experienced clinical benefits but later developed disease progression. The American Association for the Study of Liver Disease (AASLD) guidelines recommend Atezo/Bev for patients with advanced or intermediate-stage HCC, excluding those with a history of OLT or autoimmune disease. In contrast, the combination of Durva/Treme is reserved for patients at high risk for gastrointestinal bleeding [21]. TKIs remain the standard recommendation for second-line therapy, while ICPI such as Nivo/Ipi or pembrolizumab are generally considered when TKIs are contraindicated or not feasible. Nevertheless, real-world data indicate that 24.3% of patients who progressed on the Atezo/Bev regimen received ICPI as second-line treatment, highlighting the need for more evidence on their safety and efficacy [21].

Most retrospective studies on ICPI rechallenge in HCC have focused on Atezo/Bev, Nivo/Ipi, or pembrolizumab, often with TKIs administered between IO regimens [21]. In a retrospective cohort study by Wong et al., 25 patients with HCC who progressed on anti-PD-L1 therapy and were then treated with a combination of ipilimumab plus anti-PD-L1 (nivolumab or pembrolizumab). The study reported an overall response rate (ORR) of 16%, with no significant difference in outcomes between patients with primary resistance and those with acquired resistance to prior anti-PD-L1 therapy. This was the first study to demonstrate that combining anti-CTLA-4 with anti-PD-L1 therapies may help overcome resistance to previous anti-PD-L1 treatments [20].

A recently published case report described marked tumor regression following rechallenge with Durva/Treme after progression on Atezo/Bev in a patient with advanced HCC. Immunohistochemical analysis demonstrated that strong CTLA-4 blockade may redirect CD80 signaling toward CD28, therapy-enhancing T-cell activation. This mechanism may help explain the durable disease control observed in our case [22].

Based on treatment sequence and prior exposure to PD-L1-based therapy, the clinical benefit observed with Durva/Treme may be partly attributable to CTLA-4-mediated immune re-priming. The STRIDE regimen, as evaluated in the HIMALAYA trial, was specifically designed to deliver an early priming effect through a single high dose of tremelimumab followed by sustained PD-L1 blockade with durvalumab. This treatment strategy may help overcome resistance to prior PD-L1 therapy while maintaining antitumor immune activity with a favorable safety profile [13].

In the absence of a consensus on the optimal sequencing of ICPI-based therapies in advanced HCC, treatment decisions in this case were guided by guideline recommendations, prior treatment tolerance, and emerging clinical evidence. First-line Atezo/Bev was selected in accordance with the current standard of care in patients with preserved liver function. Subsequent use of TKIs was consistent with guideline-recommended second-line option after Atezo/Bev. However, both TKIs were discontinued due to poor tolerability despite dose modification.

Given the patient’s preserved performance status and prior evidence of immunotherapy responsiveness, Nivo/Ipi were subsequently initiated. Although an initial response was observed, treatment was discontinued due to grade 3–4 immune-related hepatitis. Given the complexity of liver enzyme elevations in patients with advanced HCC, formal causality assessment is essential to distinguish ICPI-induced liver injury from tumor-related hepatic dysfunction or disease progression. In this case, the episode of grade 3–4 hepatitis followed Nivo/Ipi therapy was retrospectively assessed using the updated Roussel Uclaf Causality Assessment Method (RUCAM), which is the most widely validated tool for evaluating drug-induced liver injury. The temporal relationship between ICPI exposure and liver enzyme elevation, improvement following corticosteroid therapy, and the absence of radiographic evidence of liver tumor progression at the time of hepatitis supported a probable immune-mediated drug-induced liver injury rather than tumor-related hepatic impairment. Recent studies emphasize the importance of RUCAM-based classification in ICPI-associated liver injury to avoid misclassification of drug-unrelated liver test abnormalities [23].

Given the patient’s prior response to immunotherapy and the more favorable hepatotoxicity profile of the STRIDE regimen reported in the HIMALAYA trial, Derva/Treme was selected as a rechallenge strategy, resulting in durable disease control [13].

A retrospective study by Aden et al. showed an ORR of 22% in 32 patients who received Nivo/Ipi after progression on anti-PD-L1 therapy. Scheiner et al. conducted an international multicenter retrospective analysis across 14 institutions, involving 58 HCC patients who underwent at least two lines of ICPI-based regimens. This included monotherapy, dual checkpoint inhibition, and combinations with targeted or anti-VEGF agents, with second-line therapies achieving an ORR of up 26% [20]. This finding suggests that dual ICPI therapy targeting PD-L1, and CTLA-4 may offer clinical benefits in patients unresponsive to prior anti-PD-L1 treatments, although evidence for alternative anti-PD-L1 rechallenge remains insufficient.

A retrospective study by Miura et al. evaluated the real-world efficacy and safety of Durva/Treme as a second-line treatment following Atezo/Bev in 16 advanced HCC patients. Atezo/Bev showed a higher ORR (58.3% vs. 0%) and disease control rate (DCR) (78.5% vs. 62.5%) compared to Durva/Treme. Colitis was the most common adverse event in both regimes. Liver function declined during first-line but remained stable with Durva/Treme, which was overall safer in preserving hepatic function [21]. In contrast, our case demonstrated a durable clinical response with Durva/Treme, achieving sustained disease control and AFP normalization over 39 cycles. The patient tolerated the regimen well despite prior progression and irAEs. This finding suggests that Durva/Treme may remain a valuable option as a rechallenge strategy, particularly for patients with prior irAEs, and supports its potential role in maintaining liver function. This highlights the need for prospective studies to identify patient populations most likely to benefit from ICPIs’ rechallenge strategies.

In the study by Lai et al., 65 HCC patients who failed prior anti-PD-1 therapy (mostly nivolumab or pembrolizumab) were rechallenged with durvalumab monotherapy. The ORR was 13.8% with better response in patients who had previously responded to anti-PD-1 therapy (31.3% vs. 8.7%). Median progression-free survival (PFS) and OS were 5.4 and 9.6 months, respectively. irAEs occurred in 13.8% (skin toxicity) and 7.7% (hepatitis), with no correlation between irAEs from prior anti-PD-1 and those with durvalumab. Notably, patients who experienced irAEs during durvalumab had a higher ORR [20]. Our case aligns with this finding. The patient had previously shown a clinical response to Nivo/Ipi therapy but discontinued due to immune-related hepatitis, paralleling the subgroup in the cohort who benefited from rechallenge after initial response. The patient was rechallenged with Durva/Treme and achieved SD over two years, with only oral thrush and no recurrence of autoimmune hepatitis. This favorable outcome supports the suggestion that dual ICPI may be both effective and safe in patients with prior irAES, particularly when managed carefully. This emphasizes the need for further prospective studies to better define the safety and efficacy of ICPI rechallenge strategies.

The HIMALAYA trial demonstrated that the STRIDE regimen, Durva/Treme, not only improved OS but was associated with a lower incidence of hepatotoxicity, specifically grade ≥ 3 elevation in AST (5.2%) and ALT (3.1%). Compared to higher rates observed in the CheckMate 040 trial for Nivo/Ipi, where AST and ALT elevations reached up to 16% and 8%, respectively [13]. For our patient, who had previously experienced G 3–4 autoimmune hepatitis on Nivo/Ipi, the reduced hepatotoxicity risk with Durva/Treme was a major consideration. The patient’s excellent tolerance and sustained disease control over two years further support Durva/Treme as a viable rechallenge option for advanced metastatic HCC, especially for patients with prior irAEs.

The biological success of ICPI rechallenge in this case, particularly following prior exposure to anti-PD-L1 (atezolizumab) and dual PD-1/CTLA-4 blockade (nivolumab/ipilimumab), suggests a dynamic evolution of the tumor-immune microenvironment. From a cancer biology perspective, the STRIDE regimen (single high-dose tremelimumab) may potentially induce an immune re-priming effect that differs mechanistically from traditional dosing. While prior therapies may have failed due to the expansion of exhausted T-cell phenotypes or the upregulation of alternative inhibitory pathways, the high-intensity CTLA-4 blockade may have facilitated the recruitment of additional T-cell populations from lymphoid organs.

Furthermore, the genetic landscape of HCC often involves a TMB; however, the clinical response observed here suggests that immune-related genomic features, such as HLA diversity or specific neoantigen presentation, may play a more critical role than simple mutation counts. The transition from PD-1 resistance to Durvalumab/Tremelimumab sensitivity implies a shift in the immune rheostat, CTLA-4 inhibition could potentially influence the priming phase of the cancer-immunity cycle. This observation may suggest that resistance in HCC could, in some cases, involve reversible immune dysfunction rather than permanent genetic escape, potentially providing a biological basis for sequential immunotherapy strategies. However, these proposed mechanisms remain speculative and were not directly evaluated in this case report.

While this case demonstrates a significant clinical success, several limitations must be acknowledged. First, as a single-patient case report, the findings cannot be generalized to the broader population of patients with advanced HCC. Furthermore, the clinical picture is confounded by the administration of SBRT to the right adrenal lesion during the course of Durva/Treme treatment. Although the patient achieved systemic stability, the partial response in that specific metastatic site cannot be attributed solely to the ICPI rechallenge, as localized radiation can induce both direct tumor cell death and potential abscopal effects that enhance systemic immune activity. Finally, while the patient did not experience a recurrence of grade 3/4 hepatitis, the safety of rechallenging patients with a history of severe irAEs remains a high-risk strategy that requires intensive monitoring and cannot be declared broadly safe based on this single observation.

## 4 Conclusions

This case report describes a 65-year-old patient with advanced metastatic HCC who achieved over two years of disease stability through a rechallenge with the STRIDE regimen (durvalumab and tremelimumab), despite a history of prior immunotherapy failure and grade 3/4 immune-related hepatitis. While these results are encouraging, the clinical course was likely influenced by a combination of systemic immune reactivation and localized radiotherapy. Therefore, these findings should not be interpreted as definitive evidence that ICPI rechallenge is broadly safe or effective for all patients following severe toxicity. Instead, this case demonstrates that such an approach is clinically feasible in highly selected individuals under close multidisciplinary supervision. It serves as a rationale for further prospective investigation into sequential immunotherapy strategies and the development of biomarkers to identify patients who may safely benefit from immune rechallenge.

## Data Availability

The data of this study that support our results are available upon request from the corresponding author, Abdullah Esmail (aesmail@houstonmethodist.org).

## Supplementary Materials

The supplementary material (CARE Checklist) is available online at https://www.techscience.com/doi/10.32604/or.2026.083193/s1.

## Abbreviations

The following abbreviations are used in this manuscript:

AFP	alpha-fetoprotein
ALT	alanine aminotransferase
AST	aspartate aminotransferase
BCLC	Barcelona Clinic Liver Cancer
CT	computed tomography
ECOG	Eastern Cooperative Oncology Group
HCC	hepatocellular carcinoma
ICPI	immune checkpoint inhibitor
irAEs	immune-related adverse events
MRI	magnetic resonance imaging
NCCN	National Comprehensive Cancer Network
PET	positron emission tomography
PR	partial response
SBRT	stereotactic body radiation therapy
SD	stable disease
TKIs	tyrosine kinase inhibitors

## References

[1] Ziogas IA , Evangeliou AP , Giannis D , Hayat MH , Mylonas KS , Tohme S , et al. The role of immunotherapy in hepatocellular carcinoma: A systematic review and pooled analysis of 2,402 patients. Oncologist. 2021; 26( 6): e1036– 49. doi:10.1002/onco.13638. 33314549 PMC8176986

[2] Yau T , Kang YK , Kim TY , El-Khoueiry AB , Santoro A , Sangro B , et al. Nivolumab (NIVO) + ipilimumab (IPI) combination therapy in patients (pts) with advanced hepatocellular carcinoma (aHCC): Results from CheckMate 040. J Clin Oncol. 2019; 37( 15_suppl): 4012. doi:10.1200/JCO.2019.37.15_suppl.4012.

[3] Rammohan A , Reddy MS , Farouk M , Vargese J , Rela M . Pembrolizumab for metastatic hepatocellular carcinoma following live donor liver transplantation: The silver bullet? Hepatology. 2018; 67( 3): 1166– 8. doi:10.1002/hep.29575. 29023959

[4] Qin S , Ren Z , Feng YH , Yau T , Wang B , Zhao H , et al. Atezolizumab plus bevacizumab versus sorafenib in the Chinese subpopulation with unresectable hepatocellular carcinoma: Phase 3 randomized, open-label IMbrave150 study. Liver Cancer. 2021; 10( 4): 296– 308. doi:10.1159/000513486. 34414118 PMC8339481

[5] Ranganath HA , Panella TJ . Administration of ipilimumab to a liver transplant recipient with unresectable metastatic melanoma. J Immunother. 2015; 38( 5): 211. doi:10.1097/CJI.0000000000000077. 25962109

[6] Fu XT , Qie JB , Chen JF , Gao Z , Li XG , Feng SR , et al. Inhibition of SIRT1 relieves hepatocarcinogenesis via alleviating autophagy and inflammation. Int J Biol Macromol. 2024; 278: 134120. doi:10.1016/j.ijbiomac.2024.134120. 39074701

[7] Esmail A , Rayyan Y , Rayyan H , Abdelrahim M . Evaluating standardization of neoadjuvant immunotherapy in transplant-eligible hepatocellular carcinoma populations. Jordan Med J. 2025; 59( 1): 159– 85. doi:10.35516/jmj.v59i1.3957.

[8] Chidambaranathan-Reghupaty S , Fisher PB , Sarkar D . Hepatocellular carcinoma (HCC): Epidemiology, etiology and molecular classification. Adv Cancer Res. 2021; 149: 1– 61. doi:10.1016/bs.acr.2020.10.001. 33579421 PMC8796122

[9] Abboud Y , Ismail M , Khan H , Medina-Morales E , Alsakarneh S , Jaber F , et al. Hepatocellular carcinoma incidence and mortality in the USA by sex, age, and race: A nationwide analysis of two decades. J Clin Transl Hepatol. 2024; 12( 2): 172– 81. doi:10.14218/JCTH.2023.00356. 38343612 PMC10851066

[10] Chu JH , Huang LY , Wang YR , Li J , Han SL , Xi H , et al. Pathologically successful conversion hepatectomy for advanced giant hepatocellular carcinoma after multidisciplinary therapy: A case report and review of literature. World J Gastrointest Oncol. 2024; 16( 4): 1647– 59. doi:10.4251/wjgo.v16.i4.1647. 38660668 PMC11037071

[11] Abdelrahim M , Esmail A , He AR , Khushman M , Rayyan Y . Advances in immunotherapy for transplant oncology. Cancers. 2024; 16( 13): 2369. doi:10.3390/cancers16132369. 39001431 PMC11240695

[12] Abdelrahim M , Esmail A . The use of durvalumab and tremelimumab after atezolizumab and bevacizumab in patients with hepatocellular carcinoma: Case report and literature review. Immunotherapy. 2025; 17( 11): 783– 90. doi:10.1080/1750743X.2025.2539064. 40719256 PMC12427491

[13] Abdulgader L , Esmail A . Advances in systemic therapy for unresectable hepatocellular carcinoma: Commentary on the impact of the STRIDE regimen in HIMALAYA trial. Oncol Res. 2026; 34( 3): 28. doi:10.32604/or.2026.069227. PMC12963680 41799527

[14] Özdirik B , Jost-Brinkmann F , Savic LJ , Mohr R , Tacke F , Ploner CJ , et al. Atezolizumab and bevacizumab-induced encephalitis in advanced hepatocellular carcinoma: Case report and literature review. Medicine. 2021; 100( 24): e26377. doi:10.1097/MD.0000000000026377. 34128898 PMC8213300

[15] Ishihara N , Komatsu S , Kido M , Gon H , Fukushima K , Urade T , et al. Complete response to tremelimumab plus durvalumab treatment in hepatocellular carcinoma with a bile duct tumor thrombus: A case report. Oncol Lett. 2024; 28( 1): 332. doi:10.3892/ol.2024.14465. 38807678 PMC11130751

[16] Esmail A , Hamadneh Y , Khasawneh B , Al-Rawi M , Al-Najjar E , Dhillon V , et al. Atezolizumab plus bevacizumab combination therapy in unresectable hepatocellular carcinoma: An institutional experience. Biomedicines. 2025; 13( 12): 2844. doi:10.3390/biomedicines13122844. 41462859 PMC12730803

[17] Scheiner B , Roessler D , Phen S , Lim M , Pomej K , Pressiani T , et al. Efficacy and safety of immune checkpoint inhibitor rechallenge in individuals with hepatocellular carcinoma. JHEP Rep. 2023; 5( 1): 100620. doi:10.1016/j.jhepr.2022.100620. 36578451 PMC9791167

[18] Gordan JD , Kennedy EB , Abou-Alfa GK , Beg MS , Brower ST , Gade TP , et al. Systemic therapy for advanced hepatocellular carcinoma: ASCO guideline. J Clin Oncol. 2020; 38( 36): 4317– 45. doi:10.1200/JCO.20.02672. 33197225

[19] Riley DS , Barber MS , Kienle GS , Aronson JK , von Schoen-Angerer T , Tugwell P , et al. CARE guidelines for case reports: Explanation and elaboration document. J Clin Epidemiol. 2017; 89: 218– 35. doi:10.1016/j.jclinepi.2017.04.026. 28529185

[20] Lai KC , Chen YH , Hung Y , Chiang NJ , Chen M , Chen SC . Efficacy and safety of durvalumab rechallenge in advanced hepatocellular carcinoma patients refractory to prior anti-PD-1 therapy. Hepatol Int. 2024; 18( 6): 1804– 14. doi:10.1007/s12072-024-10728-9. 39580565 PMC11632046

[21] Miura R , Ono A , Yano S , Amioka K , Naruto K , Yamaoka K , et al. Real-world efficacy and safety of durvalumab-tremelimumab as second-line systemic therapy after atezolizumab-bevacizumab in unresectable hepatocellular carcinoma. Medicine. 2024; 103( 34): e39289. doi:10.1097/MD.0000000000039289. 39288227 PMC11346847

[22] Osawa Y , Akita T , Igarashi Y , Ohtake T . Remarkable response to durvalumab and tremelimumab in a patient with hepatocellular carcinoma: A case report. Clin J Gastroenterol. 2025; 18( 6): 1144– 8. doi:10.1007/s12328-025-02223-x. 41004067

[23] Esmail A , Xu J , Burns EA , Abboud K , Sheikh A , Umoru G , et al. The impact of infections in patients treated with atezolizumab plus bevacizumab for unresectable hepatocellular carcinoma. J Clin Med. 2024; 13( 17): 4994. doi:10.3390/jcm13174994. 39274206 PMC11396642

